# Efficacy of intravitreal Lucentis injection on major and macular branch retinal vein occlusion

**DOI:** 10.1186/s12886-020-01544-4

**Published:** 2020-07-09

**Authors:** Jing Wang, Ying Li, Shu-Fen Fang, Hong Wang

**Affiliations:** 1 Department of Ophthalmology, Shandong Provincial Western Hospital, Shandong Provincial ENT Hospital, Jinan, 250023 P.R. China; 2 Department of Geriatric Medicine, Qilu Hospital, Cheeloo College of Medicine, Shandong University, Jinan, 250012 P.R. China; 3 Key Laboratory of Cardiovascular Proteomics of Shandong Province, Qilu Hospital, Cheeloo College of Medicine,Shandong University, Jinan, 250012 P.R. China; 4 Department of Ophthalmology, Laizhou People’s Hospital of Yantai, Yantai, 261400 P.R. China; 5 Department of Ophthalmology, Qilu Hospital, Cheeloo College of Medicine, Shandong University, No.107, west wenhua road, Jinan, Shandong 250012 P.R. China

**Keywords:** Lucentis, Intravitreal injection, BRVO, Major subtype, Macular subtype

## Abstract

**Background:**

The objective of our study was to assess the efficacy of intravitreal Lucentis injection on major and macular branch retinal vein occlusion (BRVO).

**Methods:**

In this retrospective analysis, 43 patients (major BRVO *n* = 24; macular BRVO, *n* = 19) were treated with intravitreal injection of Lucentis with a 1 + PRN regimen, which is diagnosed by fluorescein fundus angiography (FFA). “1 + PRN”, namely, one intravitreal injection of Lucentis at the baseline, and then continue or stop according to the condition of the patient. The following observation indexes were measured at baseline and follow-up (1–6 months): best corrected visual acuity (BCVA), foveal thickness (CFT), total retinal volume with macular diameter of 6 mm. During the follow-up, repeated injections were given according to patients’ demand, and the number of injections was recorded.

**Result:**

The observation indexes of patients with BRVO were significantly improved after 6 months of Lucentis treatment in both major and macular groups, including BCVA, CFT and the retinal volume of the 6 mm-diameter macula. Interestingly, there were significant differences in the therapeutic effect between the two groups, and the macular group had better therapeutic effect than the major group with the less number of repeated injections.

**Conclusions:**

To sum up, intravitreal injection of Lucentis was effective for both major and macular BRVO, and the efficacy in macular subtype group was better than that in major subtype group with the more obviously improvement and the less number of injections.

## Background

Retinal vein occlusion (RVO) is the second leading cause of the vascular disorders of the retina [1]. Based on the location of the occlusion, RVO can be divided into central retinal vein occlusion (CRVO) and branch retinal vein occlusion (BRVO) [2], and the incidence of BRVO is 4 times higher than that of CRVO [3]. Furthermore, Hayreh et al. proposed a new BRVO classification method that according to the location of vascular occlusion, BRVO were divided into major subtypes and macular subtypes, which showed different fundus changes [4, 5].

After RVO, capillary non-perfusion and tissue ischemia occurred due to vein occlusion, the expression of some cytokines such as vascular endothelial growth factor (VEGF) was increased, blood-retinal barrier was destroyed and vascular permeability was increased, which leads to macular edema (ME) [6, 7]. ME and retinal ischemia are the main causes of visual impairment [8]. According to statistics, the probability of secondary ME after 1 year BRVO onset is about 5–15% [9]. Hence, earlier and effective clinical treatment is essential to alleviate the symptoms of ME and restore the visual acuity of patients. To date, the related treatment mainly focuses on ME and the decrease of neovascularization after retinal vascular occlusion, and the main treatment methods include laser, steroid hormone and anti-VEGF drug injection [10].

Lucentis, also known as ranibizumab, is a humanized recombinant monoclonal antibody fragment against vascular endothelial growth factor A (VEGF-A), which can prevent choroidal neovascularization, thereby play an important role in the treatment of BRVO-induced ME [11, 12]. Up to now, studies on Lucentis have focused on the overall efficacy of anti-VEGF drugs on BRVO, and extensive reports have confirmed that intravitreal injection of Lucentis is a safe and effective treatment for BRVO [13, 14]. However, there are few studies on the efficacy of intravitreal injection of anti-VEGF drugs in different subtypes.

In this retrospective study, we collected and compared the efficacy of intravitreal injection of Lucentis in both major subtypes and macular subtypes, aiming to find out the supposing differences, and providing evidence for whether ophthalmologist should choose personal strategies based on their classification.

## Methods

### Patients

Thirty-six patients (36 eyes) with BRVO-induced ME were selected in our study, including 16 male patients and 20 female patients aged from 48 to 76. After diagnosis, patients were given “1 + PRN” strategy of intravitreal injection of Lucentis in Qilu Hospital of Shandong University between July 2016 and June 2017. “1 + PRN”, namely, one intravitreal injection of Lucentis at the baseline, and then continue or stop according to the condition of the patient. The Ethics Review Committee of Qilu Hospital, Shandong University approved this retrospective study.

Exclusion criteria: (1) former intravitreal injection; (2) refractive media: corneal opacity, cataract, vitreous turbidity; (3) other retinal or optical diseases; (4) glaucoma; (5) ocular trauma history; (6) current acute ocular affection or other active ocular diseases; (7) large non-perfusion area found in FFA examination requiring laser photocoagulation.

All patients underwent the necessary examinations before the treatment, including uncorrected visual acuity, best corrected visual acuity (BCVA), intraocular pressure (IOP), indirect ophthalmoscope, fluorescein fundus angiography (FFA), and optical coherence tomography (OCT). The patients were divided into two groups based on FFA results, including the major group (22 cases, 22 eyes) and the macular group (14 cases, 14 eyes). Majory BRVO group: The occlusion of supratemporal or infratemporal branch retinal vein trunk, involving all of their branches. Macular BRVO group: Only the branch retinal vein supplying the macular area is obstructed. The lesion is limited between superior and inferior vessel arches. The general indices of the two groups are listed in Table 1.

**Table 1:** Clinical characteristics of patients with BRVO-induced ME

characteristic	Major BRVO	Macular BRVO	t, χ^2^	P
	(*n* = 22)	(*n* = 14)		
Male	10 (50.0%)	6 (42.9%)	0.02	0.88
Female	12 (50.0%)	8 (57.1%)		
Age (years)	64.35 ± 8.30	66.19 ± 8.06	0.66	0.52
Onset Time (months)	4.60 ± 2.20	5.00 ± 2.10	0.54	0.59

*BRVO* branch retinal vein occlusion

### Intravitreal Lucentis injection

Three days before treatment, the eyes were cleaned with ofloxacin (SANTEN, OY) 4 times per day for surgery, and lacrimal passage was irrigated. Operating procedure as follows: The patient lies supine in a sterile operating room, using propmecaine hydrochloride eye drops (ALCON) for surface anesthesia. Subsequently, 0.05 ml Lucentis (10 mg/ml) was injected in the supratemporal or supranasal direction, 4 mm distance away from corneal limbus, at pars plana. After the operation, press the injection port with a sterile swab for 2 min, let out a small amount of aqueous humor via anterior chamber paracentesis and give the operating eye tobramycin and dexamethasone ophthalmic ointment (Alcon). Ofloxacin was also used for 2 weeks (4 times per day).

### Observation indices

BCVA, central foveal thickness (CFT), and volume of the 6 mm-diameter macula were collected and recorded through the same methods and instruments both before and 6 months after treatment. Simultaneously, the repeated injection case ratio and requiring injection times were recorded as well. For BCVA, we employed an international visual chart to examine it. The thickness of the defined central fovea of the macula was measured through OCT scanner(Carl Zeiss AG, Germany, Cirrus HD-OCT 4000). We also used this OCT scanner to measure the retinal volume of 6 mm-diameter macula via linear scanning of the fundus oculi.

### Follow-up

We followed the patients by month after treatment and shortened the follow-up interval if the subjective vision was found to decline. The whole follow-up period lasted for 6 months. During this time, we paid close attention to visual acuity and ME. When either of the standards below was reached, a repeated injection was needed: (1) ME increased more than 100 μm when compared with that of last time; (2) Visual acuity declined more than 1 line, and ME aggravated than last time.

### Statistical analysis

SPSS 17.0 was used to analyze the above indices in our study. BCVA was expressed in LogMAR for statistics. All data would be expressed using the form of “x ± s” if they passed the normality test. We adopted a paired t-test to compare the data before and after treatment, and took it for statistically significant when *P* ≤ 0.05.

## Result

### Patient clinical characteristics

Based on FFA, a total of 36 patients with BRVO-induced ME was divided into major and macular groups. As shown in Table 1, the major group had 22 patients, including 10 male and 12 female with the average age of 64.35 ± 8.3 years, and the mean onset time was 4.6 ± 2.2 months. Simultaneously, there were 14 patients in the macular group, including 6 males and 8 females, with an average age of 66.19 ± 8.06 years and a mean onset time of 5.0 ± 2.1. In addition, no statistical difference was found between two groups in gender, age, onset time, baseline BCVA, baseline CFT and baseline volume of the 6 mm-diameter macula.

### Baseline and follow-up BCVA

Before treatment, there was no significant difference in BCVA between the major group and macular group. (0.60 ± 0.11 vs. 0.59 ± 0.13, *P* = 0.8058). It can be seen from Table 2 that the BCVA significantly improved after 6 months Lucentis treatment compared with BCVA at baseline (*P* < 0.001), which increased to 0.28 ± 0.11 in major group and 0.21 ± 0.08 in macular group with statistically significant difference (*P* < 0.05).

**Table 2:** Comparison of observation indexes at baseline and 6 month follow-up between the major BRVO and macular BRVO

		Major BRVO(*n* = 22)	Macula BRVO(*n* = 14)	t	p
BCVA (logMAR)	baseline	0.60 ± 0.11	0.59 ± 0.13	0.25	0.81
	6 month	0.28 ± 0.11	0.21 ± 0.08	2.06	0.047
	t	9.65	9.31		
	p	< 0.001	< 0.001		
CFT (μm)	baseline	556.4 ± 117.2	538.9 ± 120.6	0.43	0.67
	6 month	271.5 ± 51.4	235.2 ± 46.1	2.15	0.039
	t	10.4418	8.8013		
	p	< 0.001	< 0.001		
The retinal volume of the 6 mm-diameter macula (mm^3^)	baseline	11.32 ± 1.13	10.88 ± 1.11	1.15	0.26
	6 month	8.91 ± 0.76	8.01 ± 0.70	3.57	0.001
	t	8.3007	8.1831		
	p	< 0.001	< 0.001		

### Baseline and follow-up CFT

The baseline CFT was 556.4 ± 117.2 μm in the major group, while it was 538.9 ± 120.6 μm in the macular group. These data did not significantly differ between two groups (*P* = 0.6685). Table 2 showed the results obtained throughout the 6 month follow-up, CFT markedly declined after 6 months Lucentis treatment compared with CFT at baseline (*P* < 0.001), and these results showed a significant difference between the major group and macular group. (271.5 ± 51.4 μm vs. 235.2 ± 46.1 μm, *P* < 0.05).

Baseline and follow-up in the retinal volume of the 6 mm-diameter macula. Throughout the 6 month follow-up, the retinal volume of the 6 mm-diameter macula decreased from 11.32 ± 1.13 mm3 to 8.91 ± 0.76 mm3 in the major group, whereas it was decreased from 10.88 ± 1.11 mm3 to 8.01 ± 0.70 mm3 in the macular group (Table 2). From the Table 2 we also can see that there was no statistically significant difference (*P* = 0.2595) between two groups before treatment, while a statistically significant difference was observed after treatment (*P* < 0.01).

### Repeated injections during follow-up

During the follow-up time, 24 patients received a repeated injection with 18 cases (81.8%) in major group, and 6 cases (42.9%) in macular group, and the difference has statistical significance between before and after treatment (χ^2^ = 5.8442, *P* < 0.05). A statistically significant difference (*P* < 0.05) was also observed in repeated injection times between two groups. In major group, the number of injections was 2.7 (P50), which was more than that in the macular group was 1.87 (P50). The number of injections in each group was presented in Supplemental Table 1.

## Discussion

In this retrospective study, we first compared the short-term effects of intravitreal Lucentis injection on patients with BROV in major group and macular group. The results showed that BCVA, CFT and the retinal volume of the 6 mm-diameter macula were significantly improved after 6 months of Lucentis treatment in both groups. In addition, the most interesting finding of the data was that there were significant differences in the therapeutic effect between the two groups, and the macular group had better therapeutic effect than the major group with the less number of repeated injections.

The main cause of vision loss in BRVO patients is BRVO-induced ME, and the presence of ME in clinic is related to the prognosis of BRVO patients [15]. The mechanism of BRVO-induced ME is generally believed to be BRVO retinal vein stenosis or occlusion, which can lead to the decrease of local retinal oxygen supply, accompanied by retinal hypoxic edema. ME will occur when the macula is involved [16]. Other studies hold different views. Previous studies have proposed that the surface area of occluded area may be directly proportional to the secretion of VEGF [2, 17]. Retinal hypoxia caused by venous occlusion can increase the expression and synthesis of VEGF, change the permeability of macular vessels, thus leading to ME [18, 19]. Furthermore, several studies have shown that VEGF was an important factor in BRVO-induced ME, and BRVO-induced ME showed significant improvement after anti-VEGF therapy [20]. Lucentis, as an anti-VEGF drug, has achieved obvious clinical efficacy for patients with BRVO [21–23]. Our data supports previous studies about the function of Intravitreal Lucentis injection in patients with BRVO. After 6 months of Lucentis treatment, BCVA increased significantly in both major and macular BRVO patients, while CFT and the retinal volume of the 6 mm-diameter macula decreased significantly, indicating that BRVO-induced ME has a significant improvement.

The natural process of BRVO depends on the location and degree of occlusion [24]. When one of the main branches of retina is blocked, it is called the major BRVO. Whereas one of the macular veins is blocked, it is called the macular BRVO. These two subtypes are obviously different in natural process and severity [2]. According to the studies of Hayreh et al. in 2015, the median time of ME subsidence was 21 months in the major BRVO group and 18 months in the BRVO group [25]*.* A retrospective study conducted by Samara WA et al. in 2016 found that in the major BRVO group, 68% of patients with mild initial defects had improved or stabilized their visual field defects, whereas the visual field defect was improved or stabilized in 85% of patients with mild initial defect in the macular BRVO group [26]. Based on the different development of the two subtypes, we investigated the influence of intravitreal Lucentis injection on major and macular BRVO, hoping to provide more targeted treatment for clinical treatment of BRVO. Our results showed that after 6 months follow-up, there was a significant difference in the treatment effect between the two groups. Observation indicators of macular group, such as BCVA, CFT and the retinal volume of the 6 mm-diameter macula, showed a more significant improvement, accompanied by less repeated injections. Previously, Noma H et al. has reported that the levels of VEGF and PlGF in the aqueous humor of patients with major BRVO were significantly higher than those of patients with macular BRVO [27]. Hence, the macular BRVO has a better response to VEGF treatment because the level of VEGF is lower than that of the main BRVO. These results suggested that intravitreal injection of Lucentis has a significant effect on BRVO patients with stable efficacy.

## Conclusion

To sum up, in our short-term observation, intravitreal injection of Lucentis was effective for both major and macular BRVO-induced ME, and there were significant differences in the efficacy of these two subtypes. The efficacy in macular subtype group was better than that in major subtype group with the more obviously improvement and the less number of injections. However, our study also had shortcomings, since patients are nonrandomized and the follow-up time was relatively short for BRVO, a highly variable disease. Hence, the longer-term observation and larger sample size are demanded to verify our results.

## Supplementary information

**Additional file 1: Table S1.** The number of injections in each group.

## Data Availability

The datasets during the current study available from the corresponding author on reasonable request.

## Abbreviations

BRVO: Branch retinal vein occlusion
FFA: Fluorescein fundus angiography
BCVA: Best corrected visual acuity
CFT: Foveal thickness
RVO: RETINAL vein occlusion
CRVO: Central retinal vein occlusion
VEGF: Vascular endothelial growth factor
ME: Macular edema
IOP: Intraocular pressure
OCT: Optical coherence tomography
