# Supplementary material for: Efficacy of intravitreal Lucentis injection on major and macular branch retinal vein occlusion
Source: BMC Ophthalmol. 2020 Jul 9;20:274. doi: 10.1186/s12886-020-01544-4 (PMC7350754; doi:10.1186/s12886-020-01544-4)
Supplement: Supplementary file 1 — Additional file 1: Table S1. The number of injections in each group. [file 12886_2020_1544_MOESM1_ESM.docx]

**Supplemental Table 1.** The number of injections in each group.

| the number of injection(s) | Major BRVO (n=22) | Macula BRVO (n=14) |
| --- | --- | --- |
| 1 | 4 | 8 |
| 2 | 10 | 3 |
| 3 | 7 | 3 |
| 4 | 1 | 0 |

P<0.05; P: the number of injections in major group vs. Macula group.
